# Caffeine Consumption and Sleep Quality in Australian Adults

**DOI:** 10.3390/nu8080479

**Published:** 2016-08-04

**Authors:** Emily J. Watson, Alison M. Coates, Mark Kohler, Siobhan Banks

**Affiliations:** 1 Centre for Sleep Research, University of South Australia, GPO Box 2471, Adelaide 5001, SA, Australia; Mark.Kohler@unisa.edu.au (M.K.); Siobhan.Banks@unisa.edu.au (S.B.); 2 Alliance for Research in Exercise, Nutrition and Activity, University of South Australia, GPO Box 2471, Adelaide 5001, SA, Australia; alison.coates@unisa.edu.au

**Keywords:** sleep hygiene, caffeine intake, sleep quantity, sleep quality, caffeine food frequency questionnaire

## Abstract

Caffeine is commonly consumed to help offset fatigue, however, it can have several negative effects on sleep quality and quantity. The aim of this study was to determine the relationship between caffeine consumption and sleep quality in adults using a newly validated caffeine food frequency questionnaire (C-FFQ). In this cross sectional study, 80 adults (M ± SD: 38.9 ± 19.3 years) attended the University of South Australia to complete a C-FFQ and the Pittsburgh Sleep Quality Index (PSQI). Caffeine consumption remained stable across age groups while the source of caffeine varied. Higher total caffeine consumption was associated with decreased time in bed, as an estimate of sleep time (*r* = −0.229, *p* = 0.041), but other PSQI variables were not. Participants who reported poor sleep (PSQI global score ≥ 5) consumed 192.1 ± 122.5 mg (M ± SD) of caffeine which was significantly more than those who reported good sleep quality (PSQI global score < 5; 125.2 ± 62.6 mg; *p* = 0.008). The C-FFQ was found to be a quick but detailed way to collect population based caffeine consumption data. The data suggests that shorter sleep is associated with greater caffeine consumption, and that consumption is greater in adults with reduced sleep quality.

## 1. Introduction

Caffeine is a widely consumed stimulant that is found in a variety of commonly consumed foods and beverages such as chocolate, soft drink (soda), tea, and coffee. Caffeine is commonly used as a fatigue countermeasure [1]. Due to its action on adenosine receptors [2,3] caffeine improves alertness [4]. The Australian Bureau of Statistics (ABS) showed that on average in 2011/2012 Australian adults aged 19–70 years had daily caffeine intakes ranging between 103 and 183 mg per day [5], with coffee being the most common source of caffeine [5]. Currently there are no consumption guidelines for caffeine in Australia. Therefore, it is important to examine the impact caffeine has on our sleep to make more informed recommendations on consumption and to better understand the impact in roles where caffeine consumption is higher.

While sleep need is individual and can differ from person to person, it is recommended that adults obtain 7–9 h per night [6]. Sleep has been shown to be important for many different cognitive and health reasons [7,8,9,10]. These benefits of sleep are not only dependent on total sleep time but also sleep quality, measured by variables such as sleep efficiency, and sleep onset latency. In adults there are a number of experimental studies investigating caffeine intake and its influence on sleep, with previous experimental studies finding that caffeine consumption can impact sleep quality [11,12]. Experimental laboratory studies have shown that when caffeine is ingested one to three hours before bedtime it decreases sleep efficiency [11,12,13], decreases total sleep time [11,12,14], and increases sleep onset latency [11,12,13]. It can also impact sleep architecture by reducing the amount of deep sleep [12,13]. However, the experimental nature of these studies does not take into account the impact of an individual’s habitual caffeine intake or sleep patterns [15].

The effect of caffeine on sleep in university and general populations has been examined by several cross-sectional surveys and/or field studies [16,17,18,19,20,21,22,23]. In these studies increased caffeine consumption has been associated with decreased total sleep time [16,21], increased naps [16], decreased time in bed [17], increased sleep efficiency due to decreased time in bed [17], daytime sleepiness [20,22], and poor subjective sleep quality [22,23]. However, in other studies increased caffeine consumption does not impact total sleep time [17], daytime sleepiness [17] or Pittsburgh Sleep Quality Index (PSQI) global score [18,19,23]. Of these cross-sectional studies, only one looked at total caffeine consumption, i.e., all caffeinated beverages and caffeinated food consumed [18], while the others examined only caffeinated beverages [16,23] or a selection of caffeinated beverages [17,19,20,21,22]. Additionally, it was not always clear what question was asked to calculate caffeine consumption, or how the participants recorded their intake. Also many studies do not consider caffeine from chocolate or examine the separate sources of caffeine individually. This is important because different age groups may prefer particular sources of caffeine. Furthermore, all the previous questionnaire studies rely on an individual knowing what drinks/foods contain caffeine. 

The mixed sleep patterns in caffeine users could be due to reporting inaccuracies. Few studies gather detailed information about caffeine consumption or sources of caffeine. To address this we have developed a caffeine food frequency questionnaire (C-FFQ) [24] that is short, does not rely on prior caffeine content knowledge, and gathers information about a wide variety of caffeine sources (coffee, tea, soft drink (soda) and chocolate beverages and foods). The overall aim of this study was to determine the relationship between caffeine consumption and sleep using this newly validated Caffeine Food Frequency Questionnaire (C-FFQ) [24] and self-reported sleep quality in adults. Specifically, this study aimed to: (1) identify what types of foods/beverages contribute to caffeine intake; (2) determine the impact of caffeine on different sleep quality variables (time in bed, sleep onset latency and sleep efficiency); and (3) determine the difference in caffeine intake between self-reported good and poor sleepers. 

## 2. Materials and Methods

This cross-sectional study was designed to investigate the relationship between habitual caffeine consumption and sleep. It was conducted between March and August 2015 at the University of South Australia, Adelaide. The project was approved by the University of South Australia’s Human Research Ethics Committee (HREC number: 30885).

### 2.1. Participants

Adults were recruited via University of South Australia web pages, social media, flyers, and word of mouth. Participants were ineligible for the study if they did not consume caffeine daily, were under the age of 18 years, not proficient in reading and writing in English, experiencing any sleep related conditions, have any conditions that affect caffeine intake, and not able to attend the University of South Australia. Participants were also excluded if they were taking any medications (prescription or over the counter, e.g., sleeping tablet, herbal supplement) to assist sleeping or alertness.

### 2.2. Procedure

All participants gave written informed consent before attending the University of South Australia. While at the University of South Australia participants were asked to complete questionnaires recalling caffeine consumption over the past week and sleep patterns over the past month.

### 2.3. Measures

Caffeine food frequency questionnaire (C-FFQ): The C-FFQ is a self-report, validated, and reliable questionnaire designed to assess the average daily caffeine consumption and the range of caffeinated products consumed [24]. The C-FFQ asks about beverages (e.g., energy drinks, soft drinks (soda), both hot and cold coffee and tea, and chocolate flavoured milk) and foods (e.g., chocolate) that were consumed in the previous week. The questionnaire requires participants to select the beverage or foods they consumed based on images of currently available products in Australia, indicating the specific brand, size, and the number of times over the last week each was consumed. Average daily total caffeine consumption and average daily caffeine amounts from beverage and food sources were expressed as mg/day.

Pittsburgh Sleep Quality Index (PSQI): The PSQI is a frequently used sleep quality questionnaire that has been shown to be reliable and valid in many populations [25,26,27]. The PSQI is a subjective measure of sleep which uses self-report to measure sleep quality and sleep disturbance over a one-month period. The PSQI contains 19 self-report questions and 5 questions rated by the bed partner or roommate. This study utilised only the self-rated questions which combine to form seven component scores [25]. The seven component scores include subjective sleep quality, sleep latency, sleep duration, habitual sleep efficiency, sleep disturbances, use of sleep medication, and daytime dysfunction. Each component has a range of 0–3 points. In all cases a score of 0 indicates the best outcome, while a score of 3 indicates the worst outcome. A global PSQI score is calculated from adding together all seven component scores to give an overall indication of sleep quality. The PSQI global score ranges from 0 to 21 points, with 0 indicating no difficulty and 21 severe difficulties. A global score of ≥5 indicates poor sleep quality [25]. This cut-off has been shown to have high specificity and sensitivity for distinguishing insomnia patients and controls [25,27]. 

### 2.4. Statistical Analysis

The C-FFQ calculates a total caffeine value and caffeine values for beverage and food subcategories as mg/day. All data were checked for normality and caffeine variables were found to be positively skewed. Log transformation of total caffeine intake allowed correction to a normal distribution; however, due to the skew of caffeine from individual beverage and food categories they could not be transformed. Data extracted from the PSQI included subscales of sleep information and from these a global score was calculated. Good sleepers were determined by a global score of less than or equal to 5 and poor sleepers were determined by a global score of above 5 as described by Buysse, Reynolds, Monk, Berman and Kupfer [25]. Sleep variables were non-normal and could not be transformed to normal.

Mean, standard deviation, median, interquartile range, and range was calculated for caffeine values to describe the population. Participants were broken into age groups similar to the Australian Bureau of Statistics categorization [5] to allow for comparison. All outliers for the sleep and caffeine variables (>3 SD above the mean) were clarified with the participant completing the questionnaire, and all variables were subsequently considered to be accurate and viable. To determine differences between groups both parametric and non-parametric tests were used. For parametric data independent samples *t*-tests were used, for the data which could not be transformed and were non normal Mann Whitney *U* tests were used, and for categorical comparisons chi square tests were used.

For further analysis, non-parametric (Spearman) correlations were utilised to assess relationships with caffeine and sleep variables. Finally, to determine if there were differences between PSQI categorical sleep variables and total caffeine consumption a one-way ANOVA were undertaken. The ANOVA dependent variables included subjective sleep quality, sleep disturbances, use of sleep medication, and daytime dysfunction. In all tests, significance was determined if *p* < 0.05.

## 3. Results

### 3.1. Study Participants

**Figure 1:**
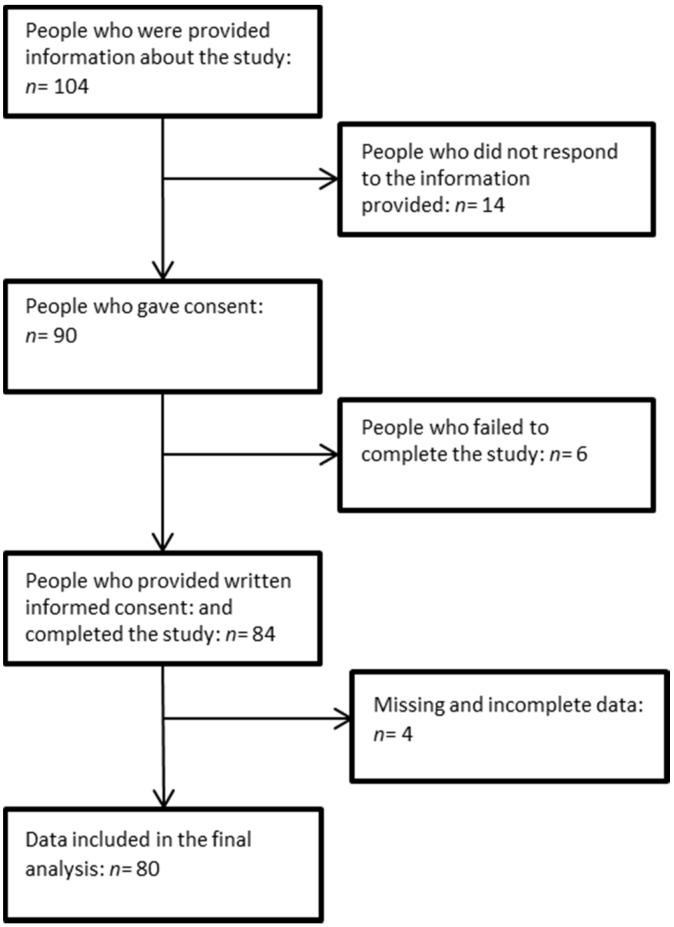
Description of participant flow throughout the study.

Of the 104 people who were provided with study information, 90 participants consented to the study and 84 participants returned the questionnaires. The final data set for analyses included 80 participants with four questionnaires having missing data (Figure 1). The final sample consisted of 54 females and 26 males, aged 19–94 years (mean ± SD 38.9 ± 19.3 years), with a mean caffeine intake of 164.9 mg/day. Most of the participants (85%) reported their sleep quality to be either very or fairly good and 86% of the participants had not taken medications to help them sleep over the past month (prescribed or over the counter). Of the sample, 80% stated that they had disturbed sleep less than once a week. Finally, 83% of the sample had minimal problems during the day due to their sleepiness (further measures of caffeine and sleep are reported in Table 1). There was no difference in age between the good sleepers (PSQI global score < 5) and poor sleepers (PSQI global score > 5) and age had no relationship with sleep efficiency (*p* = 0.0574), sleep onset latency (*p* = 0.756), however as age increased so did time in bed (*p* = 0.030).

### 3.2. Types of Foods/Beverages That Contribute to Caffeine Intake by Gender

There were no differences in total caffeine consumption between men and women *t*(78) = 0.60, *p* = 0.548. Further, Mann Whitney *U* tests were undertaken to determine any differences in caffeine consumption from different caffeine sources between genders and showed no differences in caffeine consumption from coffee, tea, soft drink and chocolate between genders. However, males consumed more caffeine from energy drinks compared with females (*U* = 589.50, *z* = −2.36, *p* = 0.018; Male median (IQR): 0.0 (0.0) mg, range: 0.0–135.0 mg; Female median (IQR): 0.0 (0.0) mg, range: 0.0–22.4 mg). Due to the small intake of energy drinks consumed overall in this sample the differences between gender in overall caffeine consumption was not considered to impact the results and males and females were analysed together.

### 3.3. Types of Foods/Beverages That Contribute to Caffeine Intake by Age

**Figure 2:**
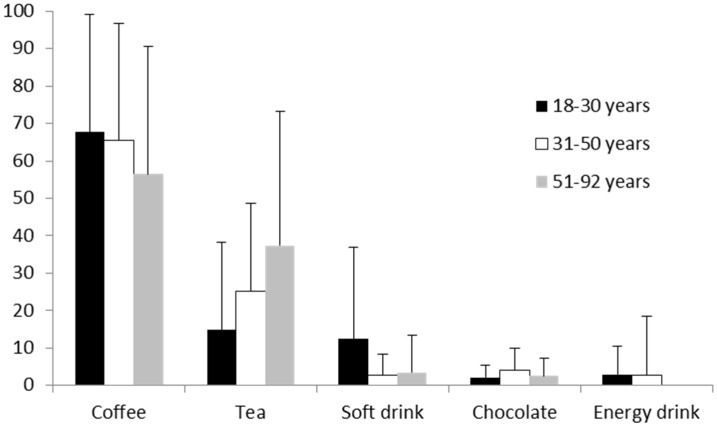
Percent of caffeine intake from different sources by age groups. Total *n* = 80; 18–30 years: *n* = 32, 31–50 years: *n =* 31, 51–92 years: *n =* 16. Notes: Chocolate includes beverages and food.

There was no significant correlation between total caffeine consumption and age (*r* = 0.167, *p* = 0.145), with similar mean intakes across age groups and a wide variation within age groups (18–30 years: 174.6 (±139.4) mg; 31–50 years: 149.1 (±74.6) mg; 51–92 years: 184.4 (±72.4) mg). Energy drink consumption significantly decreased with age (*r* = −0.297, *p* = 0.008). Tea consumption increased with age (*r =* 0.217, *p* = 0.056) and soft drink consumption decreased with age (*r* = −0.221, *p* = 0.052), however these effects were only trends. Coffee intake and chocolate intake was not correlated with age (Figure 2).

### 3.4. Impact of Caffeine on Sleep Quality

**Figure 3:**
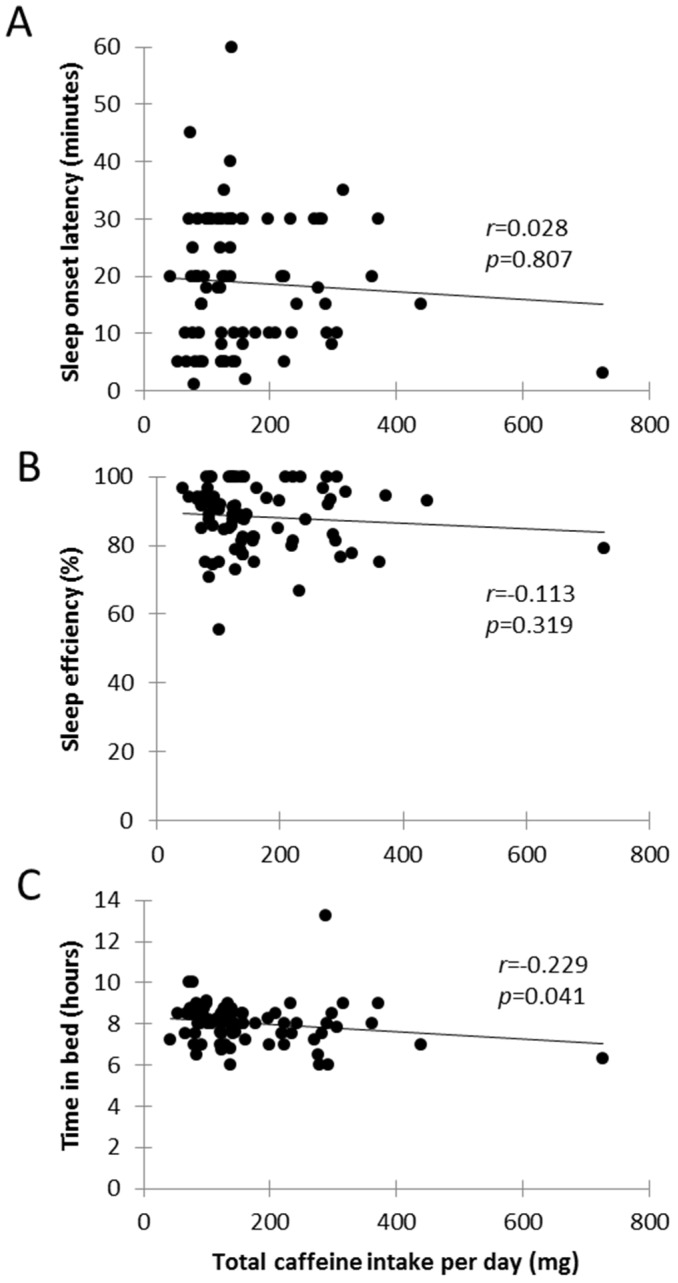
Scatterplots showing the relationship between total caffeine consumed and sleep variables: sleep efficiency, time in bed and sleep onset latency. (**A**) represents total caffeine intake versus sleep onset latency, *r* = 0.028; (**B**) shows total caffeine intake versus sleep efficiency, *r* = −0.113; (**C**) shows total caffeine intake versus time in bed, *r* = −0.229.

Sleep onset latency (Figure 3A) and sleep efficiency (Figure 3B) were not significantly correlated with caffeine (Figure 3). Time in bed was significantly related to total caffeine intake (*r* = −0.229, *p* = 0.041) with time in bed increasing with decreasing caffeine consumption (Figure 3C). Furthermore, caffeine consumption was not significantly associated with other sleep factors (subjective sleep quality, medications needed to sleep, sleep disturbance, and daily dysfunction due to sleepiness).

### 3.5. Caffeine Intake between Self-Reported Good and Poor Sleepers

To determine if caffeine consumption differed between good and poor sleepers, caffeine variables were compared between participants who reported a PSQI global score ≥ 5 (poor sleep quality) and PSQI < 5 (good sleep quality). On average, poor sleepers reported greater total caffeine consumption (mean ± SD: 192.1 ± 122.5 mg) compared to good sleepers (mean ± SD: 130.0 ± 62.6 mg; mean difference = 62.2 mg, *t*(78) = −2.73, *p* = 0.008, *d* = 0.64). 

Furthermore, caffeine from coffee intake was significantly less in good sleepers (median = 66.5, IQR = 83.1) than poor sleepers (median = 99.2, IQR = 83.3), *U* = 1028.50, *z* = 2.34, *p* = 0.019, *r* = 0.26. While caffeine from tea (*p* = 0.874), chocolate (*p* = 0.658), soft drink (*p* = 0.368) and energy drinks (*p* = 0.395) showed no differences found between groups (Table 2).

## 4. Discussion

The current study found that caffeine consumption did not change dramatically across the lifespan, with only energy drinks changing significantly across age groups. Furthermore, decreased time in bed was associated with increased caffeine consumption, and people with poor self-reported sleep quality consumed significantly more caffeine than people with good self-reported sleep quality scores.

The first aim of the study was to identify the sources of caffeine that contribute to an adult’s total caffeine intake using the newly validated C-FFQ. It was found that the average consumption of caffeine in this population was 165.1 mg per day. National data, obtained using an intensive 24 h recall process on two occasions, suggests Australian adults consume approximately 103–183 mg of caffeine per day [5]. This is similar to that found in the current study additionally, the sources of caffeine across age groups were also similar in amounts to the national data [5]. In the current study the percentage of caffeine from tea was highest in the 51–92 years age group; in the previous national data it steadily rose across the lifespan and was highest in the 71 years and over age group. Furthermore, the current sample showed that as people age they are significantly less likely to consume caffeine from energy drinks and consume less soft drinks. Finally, the percentage of caffeine from chocolate remained steady across the lifespan in both our sample and the previous national data. There was, however, a difference in percentage of caffeine from coffee between the current data and the previous national data. The current study consumed less caffeine from coffee intake in the 31–50 years age bracket when compared to the national data, with the percentage of caffeine from coffee remaining equal from 18 to 50 years and declining in the oldest age group.

The second aim of the study was to determine the relationship between caffeine and sleep quality (variables from the subscales of the PSQI). The current study showed that total caffeine consumption had a small negative correlation with time in bed. This result is similar to a previous cross sectional survey study [17] however, in contrast with Sanchez-Ortuno and colleagues [17] total caffeine consumption was not correlated with sleep efficiency. The contrast in findings could be explained by the differences in measuring caffeine. The current study used a validated food frequency questionnaire to estimate total caffeine intake and not a generic “cups per day” or “number of drinks in the last week” question as in other studies [17,18,19,23], nor did it rely on participants knowing what beverages they consume contain caffeine. Furthermore, the mean caffeine intake value is greater in the study by Sanchez-Ortuno and colleagues [17] (mean caffeine intake across the day: 225 mg) and perhaps is large enough to impact sleep compared to the current study which recorded an average of 165.1 mg per day.

The current study showed that total caffeine consumption was not correlated with subjective sleep quality, medicinal sleep aids, sleep disturbance, or daily dysfunction due to sleepiness, similar to other studies [18,23]. However, this is different to experimental laboratory studies that have found caffeine consumption negatively impacts sleep quality [11,14]. This could be explained by the dose of caffeine consumed, as laboratory studies tend to give higher amounts of caffeine to participants than the habitual amount recorded in the current study. For example Carrier et al. [13] gave 100 mg three hours before bed followed by an additional 100 mg one hour before bed time. Additionally, given that the time of caffeine consumption was not recorded in this study it is possible that it was consumed earlier in the day and therefore had less of an effect on sleep. Finally, the results of the current study indicate a global relationship between sleep and caffeine consumption and it is highly possibly that poor or short sleep drives caffeine consumption rather than the other way around. It would be beneficial for future studies to measure the timing of caffeine consumption to further investigate if habitual caffeine consumption interferes with sleep.

The third aim of the study was to determine the difference in caffeine consumption between good and poor sleepers as measured by the PSQI global score (a score which included all the variables to determine an overall score of sleep quality). There was a significant difference in caffeine consumption between participants who were classified as good sleepers and those classified as poor sleepers. Good sleepers on average consumed 67 mg less caffeine per day; approximately one cup of instant coffee. However other field studies [18,19,23] have found that the PSQI global score was not related to the amount of caffeine consumed. The contrast in findings could be explained by the differences in the ways caffeine consumption was measured as discussed above. The strength of the current study is that it measured all sources of caffeine and examined a broad population with a wide age range throughout the year, i.e., not specifically medical students [23] or students during exam period [19].

The current study provides an overview of the average daily caffeine habits of adults across the lifespan and compares it to the sleep habits. However, some limitations need to be taken into consideration. Firstly, we did not record the participants’ weight or height measurements to calculate the body mass index. The relationship between sleep and weight is well documented [8], with shorter sleep and poorer sleep quality associated with obesity. Secondly, the general population sample means that the results may not be generalizable to groups who consume more caffeine such as shift workers. Therefore, it is not possible to generalise this information to shift workers as they have different sleep and wake patterns which would potentially increase caffeine use and worsen sleep quality. Furthermore, there are limitations to self-report sleep compared to objective measures such as Actiwatches and Polysomnography. While objective measures are preferred, observational studies have widely used subjective measures previously due to cost and logistics. In adults it has been shown that subjective measures of sleep is equal to objective measures of sleep [28,29].

## 5. Conclusions

This study demonstrates the importance of accurately measuring caffeine sources as patterns of consumption can differ across the lifespan. Additionally, while this study found higher amounts of caffeine consumed related to decreased time in bed there was no association between subjective sleep quality and the amount of caffeine consumed. This could be due to the fact daily caffeine consumption was, on average, low to moderate or it could be because caffeine was not consumed close to bed time. This should be the focus of future research. With caffeine being consumed by a wide range of the population and found in many beverages and foods, it is necessary to understand the full health implications and how it may interfere with our daily behaviours such as sleep.

## Figures and Tables

**Table 1:** Caffeine consumption and sleep measures for participants.

	Mean (SD)	Median (IQR)	Range
Total caffeine (mg)	165.1 (105.3)	133.4 (120.1)	41.6–726.6
Coffee (mg)	109.9 (99.4)	87.2 (82.3)	0.0–646.6
Tea (mg)	38.0 (56.2)	13.6 (50.9)	0.0–271.4
Chocolate (mg)	3.5 (4.6)	1.7 (4.3)	0.0–20.6
Soft Drink (mg)	9.0 (24.0)	0.0 (8.6)	0.0–182.4
Energy drink (mg)	4.9 (20.4)	0.0 (0.0)	0.0–135.0
PSQI global score	5.3 (2.5)	5.0 (4.0)	0.0–21.0
SOL (min)	18.8 (11.8)	20.0 (20.0)	1.0–60.0
SE (%)	88.3 (9.3)	89.1 (13.7)	55.6–100.0
TIB (h)	8.0 (1.0)	8.0 (1.0)	6.0–13.3

Abbreviations: SD, standard deviation; PSQI, Pittsburgh Sleep Quality Index; IQR, interquartile range; mg, milligrams; SOL, sleep onset latency; SE, sleep efficiency; TIB, time in bed; global score, PSQI total score; %, percentage. Notes: Chocolate includes both food and drink.

**Table 2:** A descriptive table showing the means, standard deviations, medians, and interquartile ranges for all variables for good and poor sleepers as indicated from the PSQI. The table also shows *p* values comparing good and poor sleepers using either independent samples *t*-test. Mann Whitney *U* test and chi square tests. Participants with good sleep quality had PSQI global scores of less than 5 and participants with poor sleep quality had PSQI global scores of greater than 5.

	Good Sleep Quality *n* = 35		Poor Sleep Quality *n* = 45		*p* Values
	Mean (SD)	Median (IQR)	Mean (SD)	Median (IQR)	
Total caffeine (mg)	130.0 (62.6)	123.2 (58.2)	192.1 (122.5)	140.4 (160.6)	0.008 ^I^
Coffee (mg)	81.7 (68.5)	66.5 (83.1)	132.2 (115.0)	99.2 (83.3)	0.019 ^M^
Tea (mg)	35.4 (48.4)	13.6 (54.3)	39.5 (61.5)	13.6 (55.0)	0.874 ^M^
Chocolate (mg)	3.6 (4.0)	1.7 (4.5)	3.5 (4.9)	1.7 (4.5)	0.658 ^M^
Soft Drink (mg)	6.5 (13.2)	0.0 (5.2)	10.9 (29.2)	0.0 (10.4)	0.368 ^M^
Energy drink (mg)	2.9 (15.4)	0.0 (0.0)	6.0 (23.0)	0.0 (0.0)	0.395 ^M^
SOL (min)	11.4 (7.6)	10.0 (13.0)	24.3 (11.0)	30.0 (15.0)	0.000 ^C^
SE (%)	95.0 (4.6)	94.1 (8.6)	82.9 (8.8)	82.4 (11.1)	0.239 ^M^
TIB (h)	7.9 (0.7)	8.0 (1.3)	8.2 (1.2)	8.0 (1.1)	0.000 ^C^

Abbreviations: SD, standard deviation; PSQI, Pittsburgh Sleep Quality Index; IQR, interquartile range; mg, milligrams; SOL, sleep onset latency; SE, sleep efficiency; TIB, time in bed; global score, PSQI total score; %, percentage; ^I^, Independent samples *t*-test was used; ^M^, Mann-Whitney *U* test was used; ^C^, Chi-square test was used. Notes: Chocolate includes both food and drink.

## Abbreviations

The following abbreviations are used in this manuscript:

TST	Total Sleep Time
TIB	Time in Bed
FFQ	Food Frequency Questionnaire
PSQI	Pittsburgh Sleep Quality Index
SOL	Sleep Onset Latency
C-FFQ	Caffeine food frequency questionnaire
SE	Sleep Efficiency
